# Pancreatic Cancer-Derived Exosomes Promote the Proliferation, Invasion, and Metastasis of Pancreatic Cancer by the miR-3960/TFAP2A Axis

**DOI:** 10.1155/2022/3590326

**Published:** 2022-10-15

**Authors:** Jinhong Wu

**Affiliations:** Department of Hepatobiliary Surgery, Second Affiliated Hospital, Zhejiang University School of Medicine, Hangzhou city, 310009 Zhejiang province, China

## Abstract

**Background:**

The microRNAs (miRNAs) in cancer-derived exosomes have the ability to change tumor microenvironment. This study aims to investigate the role of miRNA in cancer-derived exosomes in pancreatic cancer (PC).

**Methods:**

Based on the analysis of PC-derived and healthy exosomes by bioinformatics analysis and quantitative real-time PCR validation, the miR-3960 was identified to be the most significantly different miRNA, and TFAP2A proved as its potential target gene. Besides, the exosomes were isolated from PANC-1 cells and identified. After that, PANC-1 cells were treated with the isolated exosomes or transfected with miR-3960 mimics or si-TFAP2A, the effect of PC-derived exosomes, as well as the miR-3960/TFAP2A axis in PC cells, were assessed by the CCK-8, EDU staining, Transwell, cell colony formation, and flow cytometry assays. Furthermore, the effects of exosomes and the miR-3960/TFAP2A axis on PC tumor growth were observed in tumor-bearing mice by the measurement of tumor weight and volume, and hematoxylin-eosin staining. Moreover, the expressions of TFAP2A/PTEN/AKT signaling proteins were detected by Western blot.

**Results:**

PC-derived exosomes were isolated successfully and proved to have promotion effects on the proliferation, metastasis, and invasion of PC cells both *in vitro* and tumor growth *in viv*o. Also, the PC-derived exosomes upregulated the TFAP2A, Bcl-2, and p-AKT/AKT protein levels, and inhibited PTEN and Bax levels and PANC-1 cell apoptosis. Overexpression of miR-3960 antagonized the promotion effect of exosomes on PC cells and the TFAP2A/PTEN/AKT signaling pathway, inhibiting the growth of tumors. Besides, si-TFAP2A enhanced the inhibitory effect of miR-3960 in PC.

**Conclusion:**

MiR-3960 antagonizes the promotion effect of tumor-derived exosomes on the proliferation, invasion, and metastasis of PC via suppressing TFAP2A.

## 1. Introduction

Pancreatic cancer (PC) has a poor prognosis and high mortality, and its incidence is rising globally [1]. It is predicted that PC will become the second cause of cancer-related death in the United States by 2030 [2]. The five-year survival rate of PC is about 6%, and the risk factors include genetics, smoking, diabetes, diet, inactivity, etc. [3]. Early PC is usually asymptomatic clinically, therefore, researchers are committed to developing markers for screening early curable pancreatic lesions [4]. It is essential to investigate the biological mechanism of the occurrence and development of PC to provide a direction for its prevention and treatment.

Exosomes are important mediators of intercellular communication, in particular, they have a strong ability to modify the tumor microenvironment [5]. Exosomes are cup-shaped in morphology under transmission electron microscopy (TEM), with diameters ranging from 40 nm to 160 nm, and carry bioactive substances, such as proteins, microRNA (miRNA), and metabolites [6]. Exosomes of host cells can activate receptors, or regulate miRNA/RNA expression in adjacent cancer cells to change their biological phenotype. For example, the miRNA of hypoxia mesenchymal stem cells-derived exosomes advanced the metastasis of lung cancer cells [7]. miRNA can transport to target cancer cells through exosomes, affecting their metastasis, invasion, and apoptosis [8]. miRNA is a kind of small non-coding RNA, which plays a role in the post-transcriptional regulation of gene expression and regulating various cellular activities [9]. For example, miR-146a-5p regulated proliferation and chemoresistance via the TRAF6/NF-*κ*B p65 signaling pathway in pancreatic ductal adenocarcinoma [10].

We screened genes by bioinformatics analysis and quantitative real-time PCR (qRT-PCR), and found the TFAP2A in PANC-1 cells was markedly inhibited by miR-3960. The transcription factor activating enhancer-binding protein 2 *α* (TFAP2A), promotes cell proliferation and apoptosis through LncRNA [11, 12]. Beck's team found that the knockout of TFAP2A decreased AKT phosphorylation in colon cancer and promoted drug resistance [13]. Gene Expression Omnibus (GEO) database is mostly used for the prediction of target miRNA [14]. So, in this study, we investigated the effects of exosomes from PC cells on the proliferation, invasion, and metastasis of PC cells based on bioinformatics analysis.

## 2. Materials and Methods

### 2.1. Screening for Differential miRNAs in Exosomes by Bioinformatics Analysis

Through GSE50632 datasets in the GEO database (https://www.ncbi.nlm.nih.gov/geo/query/acc.cgi?acc=GSE50632), the serum exosome samples of 2 patients and 2 healthy individuals were obtained in the GPL16294 platform. The original data file was analyzed by GEO2R analysis software, and the parameter *P* <0.05 and |log_2_ (FC)| >2.0 was set as the cut-off standard. The top 30 miRNAs were shown in a bar chart and the most differentially expressed miRNA was selected for this study. Genes targeted by miRNAs were predicted by the four bioinformatics algorithms, miRanda v3.3a [15], miRTargetLink (https://ccb-compute.cs.uni-saarland.de/mirtargetlink2/) [16], miRDB (http://mirdb.org/) [17] and Targetscan (https://www.targetscan.org/vert_72/) [18]. And the Gene Expression Profiling Interactive Analysis (GEPIA) [19] and quantitative real-time PCR (qRT-PCR) were used to compare the expression level of target genes in PC and normal tissues.

### 2.2. Cell Culture

The immortalized human pancreatic duct epithelial cell line, HPDE6-C7, and human PC cell lines (MIA, PANC-1, and BxPC-3 cell lines) were purchased from iCell Bioscience Inc (Shanghai, China). They were cultured with 10% fetal bovine serum (FBS) (11011-8615, Tianhang, CHN) and 90% Dulbecco's modified eagle medium (DMEM) (SH30243.01, Hyclone, US) at 37°C, 5% CO_2_.

### 2.3. The Isolation and Identification of Exosomes

The PANC-1 cells were cultured for 48 h with a medium that has been removed from exosomes by centrifugation, and the supernatant was collected. The supernatant was cleared off suspended cells and cell debris by stepwise centrifugation. After filtration by 0.22 *μ*m, exosome precipitation was obtained by centrifuging the supernatant at 120,000 g at 4°C for 2 h. The resuspended exosomes were visualized by transmission electron microscope (TEM, H-7650, Hitachi, JPN). The Nanoparticle Tracking Analysis (NTA) was carried out by a particle size analyzer (N30E, NanoFCM, Xiamen, CHN).

### 2.4. Transfection and Grouping of Cells

After constructing the plasmid expressing miR-3960 and si-TFAP2A [20, 21], according to the instructions, the PANC-1 cells with 90% confluence were transfected by Lipofectamine 2000 (11668-027, Invitrogen, US) to construct the miR-3960-overexpressed and TFAP2A-knockdown cell lines. The cells were divided into miR-NC, miR-3960, Exo + miR-NC, Exo + miR-3960, si-NC, si-TFAP2A, miR-NC + si-NC, miR-NC + si-TFAP2A, miR-3960 + si-NC, and miR-3960 + si-TFAP2A groups.

### 2.5. qRT-PCR

The supernatant was obtained from the lysed-cell suspension by centrifugation and was treated with chloroform and isopropanol successively to obtain precipitation by centrifugation, and finally, the precipitation was rinsed with 75% ethanol. The RNA was dissolved in 40 *μ*L DEPC water (R0021, Beyotime, CHN) and stored in a -80°C refrigerator. The reverse transcription was performed by HiFiScript cDNA Synthesis Kit (CW2569, cwbio, Beijing, CHN). Reaction conditions: 42°C for 15 min; and 85°C for 5 min. At last, the qRT-PCR was carried out by the SYBR Premix Ex TaqII (RR820A, Takara, JPN) according to the following procedure: 95°C for 10 min and 95°C for 15 s, then at 60°C for 60 s, performed 40 cycles. The data were processed by the relative quantitative method (2-^△△^Ct). The information on the primers was shown in Table 1.

### 2.6. Western Blot (WB)

After the lysis and centrifugation, the protein extracted from each group of cells was diluted to equal concentrations. After denaturation, the proteins were electrophoresed and the bands transferred to PVDF membranes (10600023, GE Healthcare Life, US). The blots were blocked by 5% nonfat milk and washed by TBST. Then, the membranes were incubated with the following antibodies overnight. The antibodies were anti-TSG10 (1 : 2000, DF8427), Alix (1 : 2000, DF9027), CD81 (1 : 2000, DF2306), CD9 (1 : 1000, AF5139), GAPDH (1 : 5000, AF7021), CD63 (1 : 100, AF5117), HAP70 (1 : 1000, AF5466), C-myc (1 : 2000, AF0358), Bax (1 : 2000, AF0120), Bcl-2 (1 : 1000, AF6139), TFAP2A (1 : 1000, AF0535), PTEN (1 : 1000, AF6351), p-AKT (1 : 1000, AF0016), and AKT (1 : 1000, AF6261), all purchased from Affinity Biosciences Co., Ltd, US. After washing by TBST, the membranes were incubated with corresponding secondary antibodies. The protein bands were imaged and observed by the Chemiluminescence imaging analysis system (610020-9Q) and ImageJ.

### 2.7. Cell Counting Kit-8 (CCK-8) Assay

The 96-well plates were used to culture cells in the logarithmic growth phase. At 48 h after transfection, cells and CCK-8 (HY-K0301, MedChemExpress, US) reagent (10 *μ*L/well) were incubated for 2 h in an incubator. The optical density was measured at 450 nm with the microplate reader (CMaxPlus, MD, US). The percentage of the ratio for the experimental to control groups, which is the value after subtracting separately the value of the blank-control, was used to evaluate the level of cell proliferation.

### 2.8. 5-Ethynyl-2'-Deoxyuridine (EdU) Assay

The cells with 90% confluence were cultured on 12-well plates. At 48 h after transfection, the cells were processed by the BeyoClick™ EdU Cell Proliferation Kit with Alexa Fluor 594 (C0078s, Beyotime, CHN) according to the instructions, and the slides were observed and photographed under the fluorescence inverted microscope (Ts2-FC, Nikon, JPN).

### 2.9. Transwell Assay

The Transwell chambers were coated with Matrigel that was diluted 3 : 1 in serum-free DMEM. The upper Transwell chamber (3422, Coring, US) was added with 200 *μ*L cell suspension. After 24 h, the 4% paraformaldehyde-fixed cells at the bottom chamber of the Transwell membrane were stained by the Crystal Violet Staining (548-62-9, Sunqiang, CHN). Finally, the number of deep purple cells was measured by the microscope.

### 2.10. Cell Colony Formation

1000 cells were inoculated into the 6-well plates and were incubated with 70% DMEM and 30% FBS medium. The medium was refreshed every 3 days and the colony was observed until the number of cells of each colony was greater than 50. Each well was added with 1 mL of 4% paraformaldehyde at 4°C, and the plate was rested for 60 min. And paraformaldehyde-fixed cells were cleaned gently with PBS once. Last, the cells were stained with 1000 *μ*L crystal violet for 2 min.

### 2.11. Flow Cytometry

The 1 × 10^6^ cells/mL were treated with the Annexin V FITC Apoptosis Detection Kit I (556547, BD, US), and the apoptosis rate was measured by flow cytometry (C6, BD, US).

### 2.12. Dual-Luciferase Reporter Assay

According to the instructions, the lysis cells were processed by the Dual-Luciferase Reporter Gene Assay Kit (D0010-100 T, Solarbio, CHN), and the relative light unit was measured at 560 and 465 nm with the microplate reader.

### 2.13. Xenograft Tumor Model

BALB/c male nude mice (6 weeks) were obtained from the Beijing Vital River Laboratory Animal Technology Co., Ltd (SCXK (Jing) 2016-0011). Mice were raised in a standard Specific-Pathogen-Free environment and all animal tests were approved by the Animal Experimentation Ethics Committee of Zhejiang Eyong Pharmaceutical Research and Development Center (SYXK (zhe) 2021-0033). 1 × 10^6^/100 *μ*L PANC-1 cells that were treated with blank-vector/miR-3960 plasmid or si-TFAP2A/siRNA control, were subcutaneously injected into the right groin of nude mice. The volume of tumor that was *in vivo* or resected was calculated according to the formula (Volume =0.5 × long diameter × short diameter^2^). After four weeks, the mice were euthanized, and the tumors were removed, then the size and weight were measured [22]. Then, the tumor was fixed with 10% formaldehyde and embedded in paraffin. The livers and lungs were isolated and the metastatic nodules were counted.

### 2.14. Hematoxylin-Eosin (He) Staining

After 4 *μ*m tumor paraffin slices were dewaxed and washed with distilled water, and they were stained with hematoxylin (MD911467, MDL, CHN) and eosin (613101, BaSO, CHN). Then sections were dehydrated, cleared and sealed. Finally, the microscope was used for observation. Semi-quantitative scoring (0-4 points) was performed according to the degree of inflammatory cell infiltration and the tumor cell metastatic area proportion, the extent of tumor metastasis and inflammatory cell infiltration were scored [23, 24].

### 2.15. Statistical Analysis

Statistical analysis was performed by SPSS 16.0. One-way ANOVA followed by the Turkey test was used if the multi-group data were normally distributed and conformed to the homogeneity of variance test; the paired Student's *t*-test was used if the two-group data were normal distribution but unequal variance. All data were expressed as mean ± standard deviation ($\bar{x}\pm s$), *P* <0.05 means the difference was statistically significant.

## 3. Results

### 3.1. MiR-3960 and the PANC-1 Cell Line Were Selected for the Experiment

**Figure 1:**
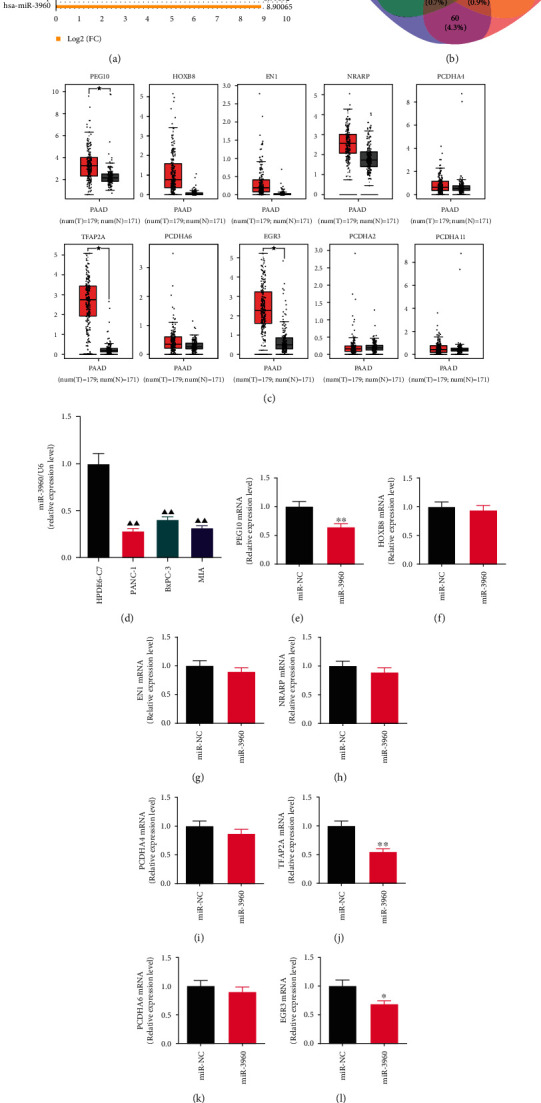
Screening of microRNAs of cancer-derived exosomes in pancreatic cancer based on bioinformatics and quantitative real-time PCR (qRT-PCR) analysis. ($\bar{x}\pm s$) (a) The bar graph shows the top 30 differentially expressed genes of GSE50632 on the GPL16294 platform using GEO2R for analysis. The Log_2_ (FC) of miR-3960 is 8.90065. (b) The Venn diagram presents the intersection of mRNAs retrieved by different databases for miR-3960. (c) The expression levels of PEG10, HOXB8, EN1, NRARP, PCDHA4, TFAP2A, PCDHA6, EGR3, PCDHA2, and PCDHA11 mRNA in PC tumor and normal groups were analyzed by the TCGA normal and GTEx data on the website of GEPIA. Red indicates the tumor group; grey indicates the normal group. (d) The miR-3960 level in HPDE6-C7, PANC-1, BxPC-3 and MIA cell lines by qRT-PCR analysis (*n* =3). (e-n) The effect of miR-3960 on the levels of PEG10, HOXB8, EN1, NRARP, PCDHA4, TFAP2A, PCDHA6, EGR3, PCDHA2, and PCDHA11 mRNA by qRT-PCR (*n* =3). ^★^*P* <0.01 compared to the normal group; ^▲▲^*P* <0.01 compared to the HPDE6-C7 group; ^∗^*P* <0.05, ^∗∗^*P* <0.01 compared to the miR-NC group.

The bioinformatics analysis results were shown in Figures 1(a)–1(c). The miR-3960 was the most significantly different miRNA in tumor and normal tissues, and the log_2_(FC) is 8.90065 (Figure 1(a)). Combined with four platform analyses and literature review, we obtained ten potential target genes (Figure 1(b)). They are PEG10, HOXB8, EN1, NRARP, TFAP2A, PCDHA6, EGR3, PCDHA2, PCDHA4, and PCDHA11. Based on TCGA normal and GTEx database, the expressions of these genes in PC were analyzed, and the levels of PEG10, TFAP2A and EGR3 was remarkably greater in tumor compared to normal group (*P* <0.01) (Figure 1(c)).

In order to determine the PC cell lines used for the experiments, the expression of miR-3960 in HPDE6-C7, PANC-1, BXPC-3, and MIA cell lines was analyzed by qRT-PCR (Figure 1(d)). The miR-3960 expression levels in PANC-1, BXPC-3, and MIA cell lines were immensely lower in comparison to it in HPDE6-C7 cell line (*P* <0.01), and the level in PANC-1 was the lowest. Thus, PANC-1 cell line was used for subsequent experiments.

The expression levels of PEG10, HOXB8, EN1, NRARP, PCDHA4, TFAP2A, PCDHA6, EGR3, PCDHA2, and PCDHA11 mRNA were measured by qRT-PCR in PANC-1 cells transfected with miR-3960 mimics. Among them, in the miR-3960 group, the PEG10, TFAP2A, and EGR3 mRNA levels were markedly inhibited in comparison with the miR-NC group, with TFAP2A as the most significant one (*P* <0.01, *P* <0.05) (Figures 1(e), 1(j), and 1(l)). The mRNA levels of HOXB8, EN1, NRARP, PCDHA4, PCDHA6, PCDHA2, and PCDHA11genes were not significantly different (*P* >0.05) (Figures 1(f)–1(i), 1(k), 1(m) and 1(n)).

### 3.2. Identification of Exosomes in PANC-1

**Figure 2:**
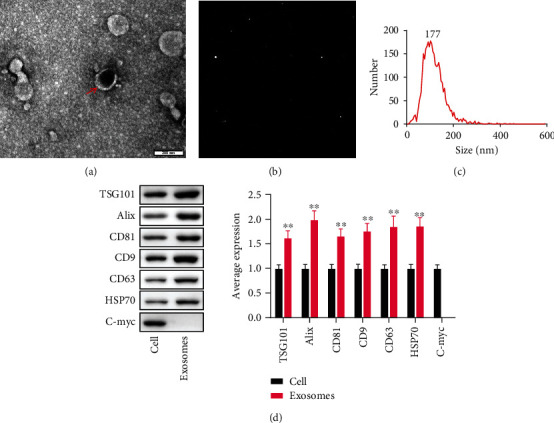
The identification of PANC-1-derived exosomes. ($\bar{x}\pm s$, *n* =3). (a) Transmission electron microscopy. (Scale bar =200 nm) (b and c) Nanoparticle Tracking analysis. (d) The average levels of TSG101, Alix, CD81, CD9, CD63, HSP70 and C-myc. ^∗∗^*P* <0.01 compared to the cell group.

The TEM and NTA analyses were performed to identify the exosomes in PANC-1. The morphology of exosomes with typical bilayer structures in the form of discs- or cup-shaped was observed by TEM (Figure 2(a)), suggesting the high purity of the samples. And the NTA analysis showed that the exosomes were spherical in shape and the average diameter is 177 nm, the size range is 12-300 nm, with a relatively normal distribution (Figures 2(b) and 2(c)).

WB indicated the presence of typical exosome marker proteins in exosomes (Figure 2(d)). In the exosome group, the TSG101, Alix, CD81, CD9, CD63, and HSP 70 protein expression levels were remarkably greater in comparison to the cell group (*P* <0.01). And the nuclear protein C-myc was not expressed in exosomes.

### 3.3. The Effect of Tumor-Derived Exosomes on the Proliferation, Invasion, and Metastasis of PC in BALB/C Nude Mice

**Figure 3:**
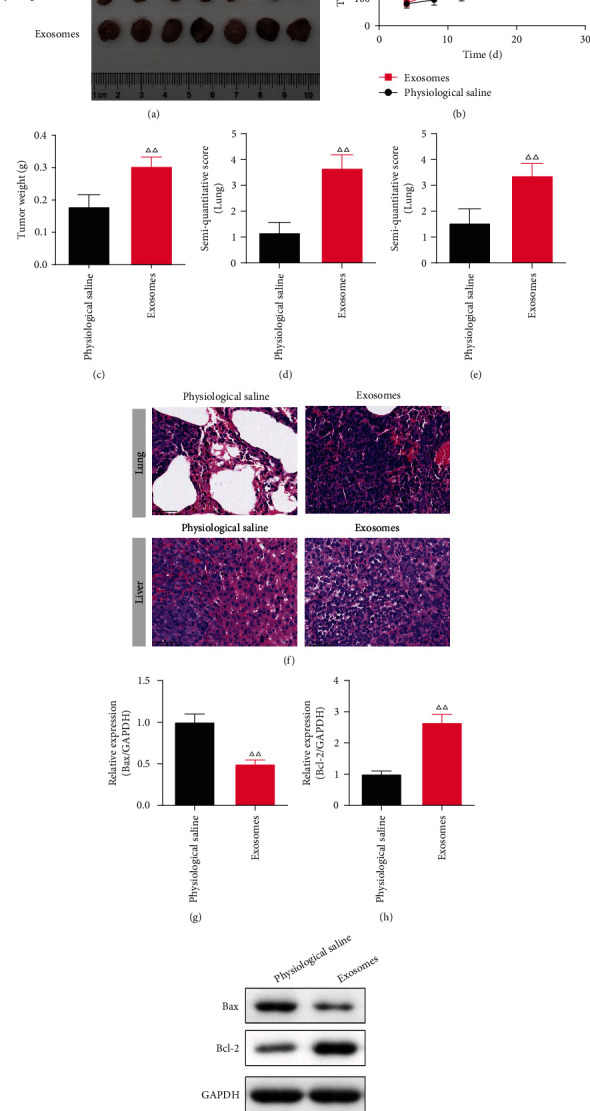
The effect of tumor-derived exosomes on pancreatic cancer (PC) on the xenograft tumor model. ($\bar{x}\pm s$, *n* =7) (a) The photographs of tumors that were dissected from PC tumor bearing mice every fourth day. (b) The tumor volume. (c) The tumor weight. (d-f) The semi-quantitative score and typical picture of Hematoxylin-eosin (HE) staining in the lung and liver. (×400) (g-i) The Bax and Bcl-2 protein levels in the lung of PC mice (*n* =3). *^ΔΔ^P*<0.01 compared to the cell group.

The xenograft tumor model was established to observe the proliferation of PC. Compared with the physiological saline group, the volume and weight of the tumor were markedly greater in the exosomes group (*P* <0.01). (Figures 3(a)–3(c)).

The invasion and metastasis of PC were observed by the HE staining. The morphology of the lung in mice of the physiological saline group was clear and complete, and there were no tumor metastases. While in the exosomes group, there was marked tissue expansion with thickening of the alveolar septa and permeation of inflammatory cells in the lung. And the semi-quantitative score was greater in the exosomes group in comparison with the physiological saline group (*P* <0.01) (Figures 3(e) and 3(f)). Similarly, in the exosomes group, the liver was fragmented and not visible in morphology, with prominent inflammatory cell infiltration and increased metastatic foci, and the semi-quantitative score was higher in comparison with the physiological saline group (*P* <0.01) (Figures 3(e) and 3(f)).

The Bax and Bcl-2 proteins in the liver of exosomes treatment mice were tested by WB. The Bax protein level of the exosomes group was immensely decreased in comparison to the physiological saline group (*P* <0.01), and the level of Bcl-2 protein was the opposite (*P* <0.05). (Figures 3(g)–3(i)).

### 3.4. The Effect of Tumor-Derived Exosomes in Proliferation, Metastasis, Invasion and Apoptosis in miR-3960-Overexpressed PANC-1 Cells

**Figure 4:**
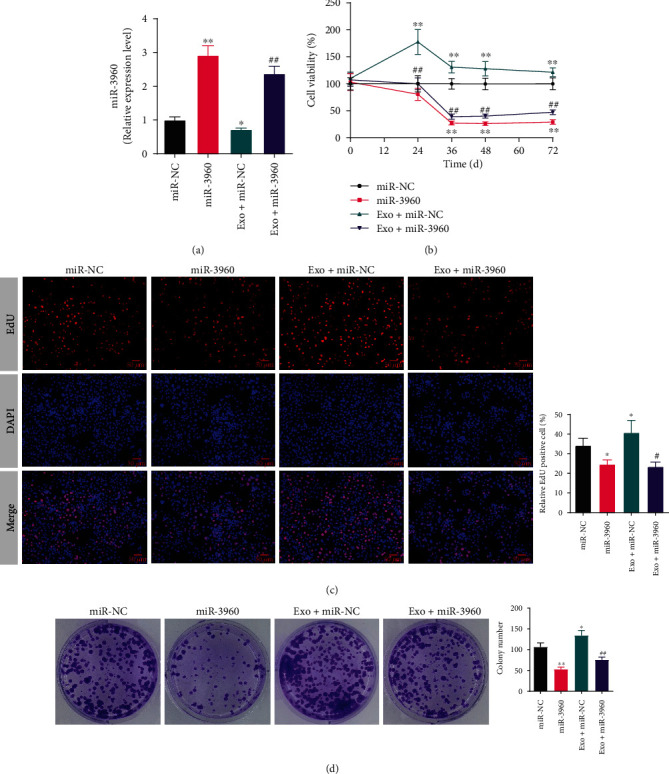
The effect of tumor-derived exosomes on the proliferation in miR-3960-overexpressed PANC-1 cells. ($\bar{x}\pm s$) (a) The quantitative real-time PCR result of miR-3960 (*n* =3). (b) The cell viability analyzed by Cell Counting Kit-8 (*n* =5). (c) The typical pictures and relative positive cells of 5-Ethynyl-2'-deoxyuridine (EdU) assay (*n* =3). Red indicates EdU positive cells and blue indicates DAPI positive cells. (×400) (d) The typical pictures and colonies number of cell colony formation assay (*n* =3). ^∗^*P* <0.05, ^∗∗^*P* <0.01 compared to the miR-NC group, ^#^*P* <0.05, ^##^*P* <0.01 compared to the Exo + miR-NC group.

The inhibition of exosomes on the miR-3960 expression level was examined by qRT-PCR. In comparison to the miR-NC group, the level in the miR-3960 group was markedly raised (*P* <0.01), and in the Exo + miR-NC was the opposite. Comparing the Exo + miR-3960 group with the Exo + miR-NC group, the miR-3960 level was notably raised (*P* <0.01) in miR-3960 group, suggesting that exosome treatment can significantly inhibit miR-3960 expression (Figure 4(a)).

To analyze the effect of exosome on miR3960-overexpressed PANC-1 cell proliferation, we carried out the CCK-8, EdU, and cell colony formation assays (Figures 4(b)–4(d)). The viability of PANC-1 cells in the miR-3960 group was notably repressed in comparison to the miR-NC group (*P* <0.01). On the contrary, in the Exo + miR-NC group, the viability was notably raised in comparison with the miR-NC group (*P* <0.01). Additionally, the viability of cells was repressed significantly in the Exo + miR-3960 group compared to the Exo + miR-NC group. The results of EdU and cell colony formation analysis was in consistent with the CCK-8 results (Figures 4(c)–4(f)). In comparison with the miR-NC group, the EdU positive cells and relative colony number were inhibited in the miR-3960 group and was raised in the Exo + miR-NC group (*P* <0.05, *P* <0.01). In the Exo + miR-3960 group, the percentage of EdU positive cells was remarkably lower compared to the Exo + miR-NC group (*P* <0.05, *P* <0.01).

**Figure 5:**
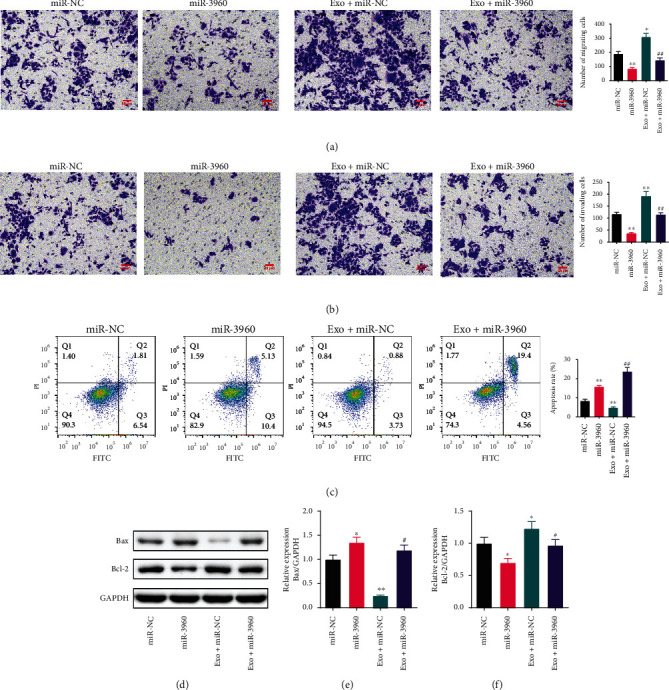
The effect of tumor-derived exosomes on the migration, invasion and apoptosis in miR-3960-overexpressed PANC-1 cells. ($\bar{x}\pm s$, *n* =3) (a) The migration of PANC-1 cells tested by Transwell assay. (×200) (b) The invasion of PANC-1 cells tested by Transwell assay. (c) The apoptosis of PANC-1 cells tested by flow cytometry. (d-f) The Bax and Bcl-2 protein levels measured by Western blot. ^∗^*P* <0.05, ^∗∗^*P* <0.01 compared to the miR-NC group, ^#^*P* <0.05, ^##^*P* <0.01 compared to the Exo + miR-NC group.

The abilities of PANC-1 cell metastasis and invasion was tested by the Transwell assay (Figures 5(a) and 5(b)). Compared to the miR-NC group, the numbers of migrating and invading cells were notably reduced in the miR-3960 group (*P* <0.01). And the cells were both markedly increased in the Exo + miR-NC group compared with the miR-NC group (*P* <0.05 or *P* <0.01). In the Exo + miR-3960 group, the numbers of migrated and invaded cells were repressed significantly in comparison to the Exo + miR-NC group (*P* <0.01).

The apoptosis levels in PANC-1 cells were inversely correlated with the viability of the cells (Figure 5(c)). The apoptotic rate was notably enhanced in the miR-3960 group and was immensely reduced in the Exo + miR-NC group, both in comparison to the miR-NC group (*P* <0.01). The percentage of apoptotic cells in the miR-3960 exosome treatment cells had a marked increase (*P* <0.01).

Tumor-derived exosomes regulated the expression of Bax and Bcl-2 proteins in PANC-1 via miR-3960. We measured the expression of Bax and Bcl-2 in PANC-1 by WB. The Bax expression was markedly increased in the miR-3960 group in comparison to the miR-NC group (*P* <0.05). In the Exo + miR-NC group, the Bax protein was immensely reduced compared to the Exo + miR-3960 group (*P* <0.01). The Bax protein level was notably greater in the Exo + miR-3960 group than in the Exo + miR-NC group (*P* <0.01). The expression of Bcl-2 was opposite to that of Bax (*P* <0.05). (Figures 5(d)–5(f)).

### 3.5. The Effect of Tumor-Derived Exosomes on the Proliferation, Invasion, and Metastasis of miR-3960-Overexpressing Pancreatic Cancer in BALB/C Nude Mice

**Figure 6:**
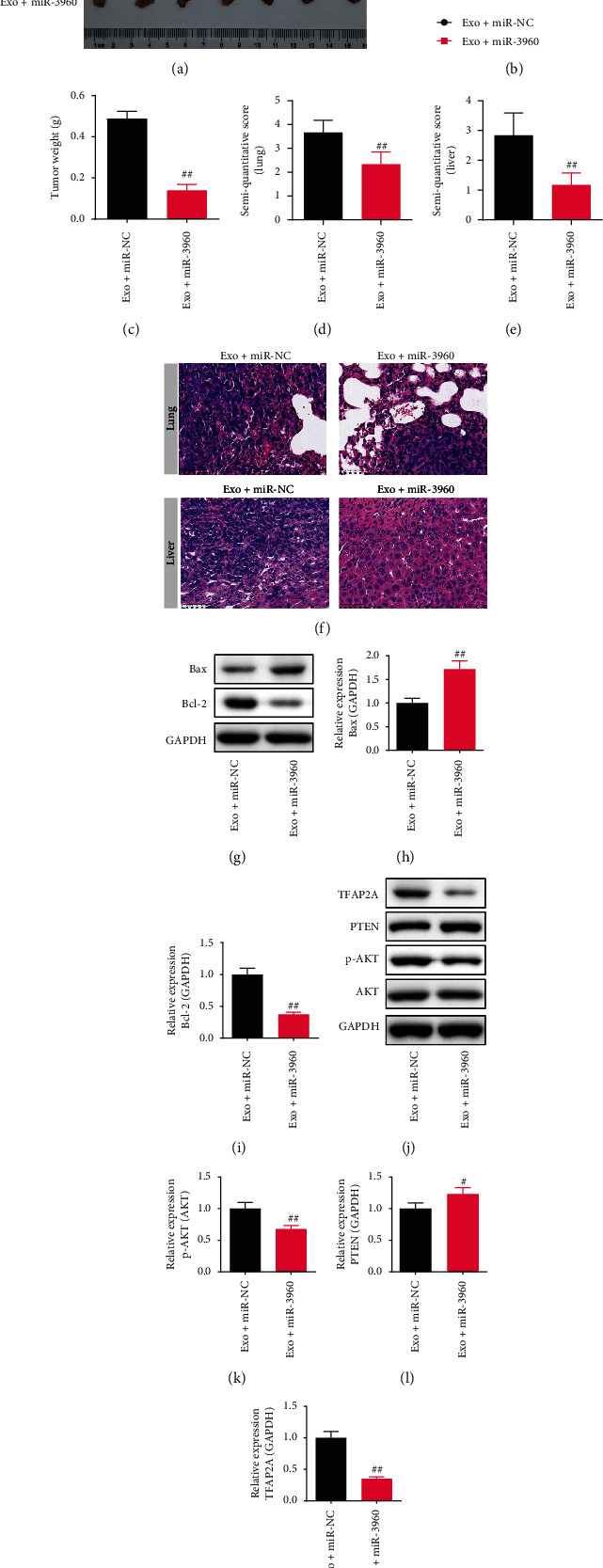
The effect of tumor-derived exosomes on miR-3960-overexpressed pancreatic cancer (PC) on the xenograft tumor model. ($\bar{x}\pm s$, *n* =7) (a) The photographs of tumors dissected from PC mice. (b) The tumor volume. (c) The tumor weight. (d-f) The semi-quantitative score and typical picture of Hematoxylin-eosin staining in the lungs and livers. (×400) (g-i) The Bax and Bcl-2 protein levels in the lungs of PC mice. (j-m) The p-AKT/AKT, PTEN, TFAP2A levels in the lungs of PC mice (*n* =3). ^##^*P* <0.01 compared to the Exo + miR-NC group.

Compared to the Exo + miR-NC group, the volume and weight of the tumors were notably decreased in the Exo + miR-3960 group (*P* <0.01) (Figures 6(a)–6(c)). The HE staining was performed to observe the invasion and metastasis of PC. Compared to the Exo + miR-NC group, the morphology of the lung and liver in the Exo + miR-3960 group were complete and clear, and the tumor metastases were few. The semi-quantitative score of the lung and liver were lower in the Exo + miR-3960 group in comparison with the Exo + miR-NC group (*P* <0.01) (Figures 6(d)–6(f)).

The Bax, Bcl-2, TFAP2A, PTEN, and p-AKT/AKT proteins expression in the lung were observed by WB. The Bax and PTEN protein levels in the Exo + miR-3960 group were immensely increased in comparison to the Exo + miR-NC group (*P* <0.05), and the Bcl-2, TFAP2A, and p-AKT/AKT expression decreased (*P* <0.01). (Figures 6(g) and 6(h)).

### 3.6. The Effect of the TFAP2A-Knockdown on the Proliferation, Invasion, and Metastasis of Pancreatic Cancer in BALB/C Nude Mice

**Figure 7:**
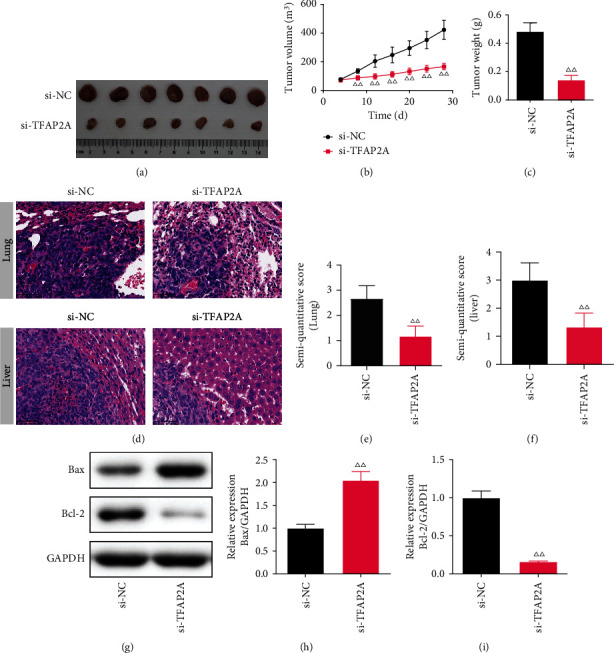
The effect of TFAP2A-silencing on pancreatic cancer. ($\bar{x}\pm s$) (a) The photographs of tumors dissected from PC mice. (*n* =7) (b) The tumor volume. (*n* =7) (c) The tumor weight. (*n* =7) (d-f) The semi-quantitative score and typical picture of Hematoxylin-eosin staining in the lung and liver. (×400, *n* =7) (g-i) The Bax and Bcl-2 protein levels in the lung of PC mice (*n* =3). *^ΔΔ^P*<0.01 compared to the si-TFAP2A group.

Based on the results of our bioinformatics and literature analysis, we investigated TFAP2A in PC. We silenced TFAP2A *in vivo* to observe the tumor proliferation and invasion. The volume and weight of the tumor were both notably decreased in the si-TFAP2A group (*P* <0.01) (Figures 7(a)–7(c)). In comparison to the si-NC group, the lung and liver morphology in the si-TFAP2A group were normal and complete, and the tumor metastases were less. The semi-quantitative score of the lung and liver were both decreased in the TFAP2A-silenced PC mice compared to the si-NC group (*P* <0.01) (Figures 7(d)–7(f)).

In the liver, the expression of Bax and Bcl-2 proteins in TFAP2A-silencing mice were measured by WB. In the si-TFAP2A group, the Bax protein level was notably increased (*P* <0.01, *P* <0.05), while the Bcl-2 level was decreased (*P* <0.01) in comparison with the si-NC group (Figures 7(g)–7(i)).

### 3.7. The Effect of TFAP2A-Knockdown on miR-3960-Overexpressed PANC-1 Cells

**Figure 8:**
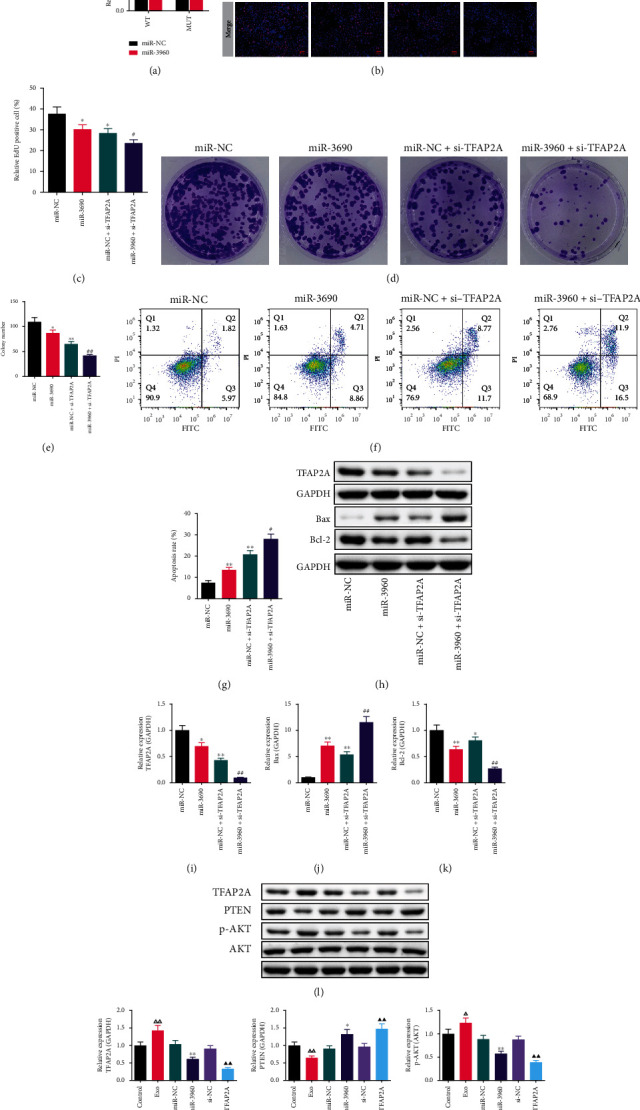
The effect of TFAP2A-knockdown on miR-3960-overexpressed PANC-1 cells and the expression of TFAP2A and PTEN/Akt signaling proteins in PANC-1 cells. ($\bar{x}\pm s$, *n* =3) (a) The dual-luciferase reporter assay. (b-c) The typical pictures and relative positive cells of EdU assay (*n* =3). Red indicates EdU positive cells and blue indicates DAPI positive cells. (×400) (d, e) The typical pictures and colonies number of cell colony formation assay. (f, g) The apoptosis of PANC-1 cells tested by Flow cytometry. (h-k) The TFAP2A, Bax and Bcl-2 protein levels in miR-3960-overexpressed PANC-1 cells. (l-o) The TFAP2A, PTEN, p-AKT/AKT protein levels. ^∗^*P* <0.05, ^∗∗^*P* <0.01 compared to the miR-NC group, ^##^*P* <0.01 compared to the miR-NC + si-TFAP2A group, *^ΔΔ^P*<0.01 compared to the control group, ^▲▲^*P* <0.01 compared to the si-NC group.

We investigated the interaction of miR-3960 and TFAP2A through the dual-luciferase reporter assay. The luciferase activity of the miR-3960 transfected cells was markedly decreased in the WT group compared to the miR-NC group, while this decrease was blocked by mutation of the 3 ‘UTR plasmid sequence of the TFAP2A gene (Figure 8(a)).

The proliferation of PANC-1 cells was measured by EdU (Figures 8(b) and 8(c)) and cell colony formation assays (Figures 8(d) and 8(e)). The relative proportion of EdU positive cells and colony number were notably lower in the miR-3960 and miR-NC + si-TFAP2A group than those in the miR-NC group (*P* <0.05, *P* <0.01). The number of EdU positive cells and cell colonies in the miR-3960 + si-TFAP2A group was significantly reduced compared to the miR-NC + si-TFAP2A group (*P* <0.05, *P* <0.01).

The flow cytometry was used to test the apoptosis in PANC-1 cells. The proportion of apoptotic cells in the miR-3960 and miR-NC + si-TFAP2A groups was markedly higher compared to the miR-NC group (*P* <0.01). Also, comparing the miR-3960 + si-TFAP2A group with the miR-NC + si-TFAP2A groups, the proportion of apoptotic cells was markedly raised in the miR-3960 + si-TFAP2A groups (*P* <0.05). (Figures 8(f) and 8(g)).

In addition, the TFAP2A, Bax, and Bcl-2 protein in the PANC-1 cells were measured by WB (Figures 8(h)–8(k)). In comparison to the miR-NC group, the TFAP2A level was reduced in the miR-3960 and miR-NC + si-TFAP2A groups (*P* <0.05 or *P* <0.01). Similarly, the TFAP2A level in the miR-3960 + si-TFAP2A group was notably decreased in comparison to the miR-NC + si-TFAP2A group (*P* <0.01). (Figure 8(i)).

In the miR-3960 group, the Bax protein level was immensely greater than it in the miR-NC group (*P* <0.01), while the Bcl-2 expression level was lower compared to the miR-NC group (*P* <0.01). In the miR-NC + si-TFAP2A group, the level of Bax protein was markedly increased and the Bcl-2 protein was immensely reduced, compared to the miR-NC group (*P* <0.01 or *P* <0.05). The Bax protein was markedly greater and the Bcl-2 protein was notably lower in the miR-3960 + si-TFAP2A group in comparison to the miR-NC + si-TFAP2A group (*P* <0.01 or *P* <0.05). (Figures 8(j) and 8(k)).

### 3.8. WB Analysis on the Expression of TFAP2A and PTEN/Akt Signaling Proteins in PANC-1 Cells

The TFAP2A and PTEN/Akt signaling proteins in the miR-3960-overexpressed and TFAP2A-silenced PANC-1 cells were measured (Figures 8(l)–8(o)). In comparison with the control group, the expression TFAP2A and p-AKT/AKT was markedly raised in the Exo group (*P* <0.01, *P* <0.05), while the PTEN level was notably decreased (*P* <0.01). The TFAP2A and p-AKT/AKT levels in the miR-3960 group were reduced in comparison to the miR-NC group (*P* <0.01). Conversely, the PTEN protein level was raised significantly (*P* <0.05). The expression of TFAP2A and p-AKT/AKT was markedly decreased and the PTEN protein was markedly higher in the TFAP2A-silenced cells than that in the control cells (*P* <0.01).

## 4. Discussion

Exosomes are mediators of signal and molecular transmission between cells. By secreting exosomes, cells participate in a variety of biological activities [25]. In tumors, exosomes play a major role in the formation and development of different cancers [26]. It was found that exosomes were involved in liver metastasis of PC by activating the TGF-*β* in PC mouse model [27]. Therefore, the study on the mechanism of bioactive substances in exosomes of PC is helpful to understand the occurrence, development, and metastasis of PC. In this study, we used miR-3960 plasmid transfection in PANC-1 cells and the xenograft tumor model in BALB/C nude mouse to study the biological mechanism of miR-3960 in the exosomes of PC cells. For the first time, we demonstrated that miR-3960 in exosomes of PC inhibits tumor proliferation, metastasis, and invasion via TFAP2A.

We found that there was a big difference in miR-3960 expression between patients with PC and healthy individuals. Mo proved that knockdown of miR-3960 antagonized the apoptosis of small-cell lung cancer H446 cells induced by Caffeic Acid Phenethyl Ester [28]. While Yang's team believed that miR-3960 promoted drug resistance in head and neck squamous cell carcinoma [29]. MiR-3960 has different biological effects on different kinds of tumor cells. This study examined the changes in the miRNA spectrum in exosomes derived from PC and revealed the antitumor effect of miR-3960, that overexpression of miR-3960 could inhibit the proliferation, metastasis, and invasion of PC cells and promote apoptosis. In PC cells, the low expression of miRNA 3960 may be a biological marker of abnormal proliferation, metastasis and invasion.

In recent years, TFAP2A has been proved to promote the proliferation and metastasis of lung adenocarcinoma, cervical cancer, and other cancer cells, mainly by affecting EMT to promote tumor metastasis [30, 31]. This study demonstrated that knockdown of TFAP2A could block the apoptosis of PC cells, and this process involved the regulation of apoptosis-related proteins. In this study, Bcl-2, as an anti-apoptotic protein [32], was significantly down-regulated after overexpression of miR-3960, and this effect could be achieved by the knockdown of TFAP2A. The raise of Bax, the pro-apoptotic protein, further prove the role of miR-3960 in promoting tumor cell apoptosis. Exosomes may antagonize PC cell apoptosis and promote its proliferation by inhibiting the expression of miR-3960. Research reported that the effect of miR-3196 on the JAK2/STAT3 pathway by TFAP2A [33], and the JAK2/STAT3 pathway was proved that was related with apoptosis [34]. This may be a relevant biological mechanism for the regulation of Bax and Bcl2 by TFAP2A and deserves further exploration.

In addition, we explored the expression of molecules in the PTEN/Akt signaling pathway. PTEN/AKT is a pathway related to tumor metastasis. Cheng et al. discovered that low PTEN expression was related to the poor survival in PC patients [35]. Moreover, a study has proved that the exosomes of high metastatic liver cancer cells inhibited PTEN to activate the Akt signaling pathway and promoted the EMT and metastasis of cancer cells [36]. Our study found that PC-derived exosomes inhibited PTEN and activated Akt, while miR-3960-overexpression and TFAP2A-knockdown antagonized this effect, suggesting that the inhibitory effect of miR-3960-overexpressed on PC cell metastasis by inhibiting TFAP2A may be closely related to the regulation PTEN/Akt signaling pathway. This signaling pathway may be one of the key biological mechanisms for PC cells-derived exosomes to advance tumor metastasis, and invasion. Further studies are worth unfolding in the future.

## 5. Conclusions

In summary, this study investigated miR-3960-containing exosomes in regulating the proliferation, metastasis and invasion of PANC-1cells. By constructing the xenograft tumor model, the promoting effect of exosomes on tumor proliferation, metastasis and invasion was observed and verified. Overexpression of miR-3960 can antagonize this effect by inhibiting TFAP2A. This study proved that the inhibition of miR-3960 on PC via the TFAP2A/PTEN/AKT pathway and provides the ideas and methods to study the biological mechanism of exosomes in promoting tumor development and treatment of PC.

## Figures and Tables

**Table 1:** Primer sequences.

Gene	Forward primer (5'-3')	Reverse primer (3'-5')
Human miR-3960	GGCGGCGGCGGAGGCGGG	CAGTGCGTGTCGTGGAGT
Human U6	AAAGCAAATCATCGGACGACC	GTACAACACATTGTTTCCTCGGA
Human PEG10	AGCAGTCGGAGGAGAACAAC	CACTGGGCCATGAAAGGAG
Human HOXB8	GTCCCTGCGCCCCAATTATTA	GCCCGTGGTAGAACTCCTG
Human EN1	CCCGAGTGTTTCACAGCCAAT	GGATGCTGGAAAGTCTCGTTC
Human NRARP	TCAACGTGAACTCGTTCGGG	ACTTCGCCTTGGTGATGAGAT
Human TFAP2A	AGGTCAATCTCCCTACACGAG	GGAGTAAGGATCTTGCGACTGG
Human PCDHA6	GGAAAGCAATGTCTGCTCCTC	CCTCCTCGGGTACGGAGTAG
Human EGR3	GACATCGGTCTGACCAACGAG	GGCGAACTTTCCCAAGTAGGT
Human PCDHA2	AGCGCGGAATGTAGCATCC	AGCAGCCTTGATTCGGGAAAC
Human PCDHA4	ACCTGTCCATCGCGGAATC	CAAGACCTTTTACCAGCTCGTC
Human PCDHA11	GCAATCGGACTCGCGTTTTC	GCCTGTAGGTCAATAGAGCATTC
Human GAPDH	GGAGCGAGATCCCTCCAAAAT	GGCTGTTGTCATACTTCTCATGG

## Data Availability

The data used to support the findings of this study are available from the corresponding author upon request.

## References

[1] Klein A. P. (2021). Pancreatic cancer epidemiology: understanding the role of lifestyle and inherited risk factors. *Nature Reviews Gastroenterology & Hepatology*.

[2] Siegel R. L., Miller K. D., Jemal A. (2017). Cancer statistics, 2017. *CA: A Cancer Journal for Clinicians*.

[3] Ilic M., Ilic I. (2016). Epidemiology of pancreatic cancer. *World Journal of Gastroenterology*.

[4] Vincent A., Herman J., Schulick R., Hruban R. H., Goggins M. (2011). Pancreatic cancer. *The Lancet*.

[5] Zhang L., Yu D. (2019). Exosomes in cancer development, metastasis, and immunity. *Biochimica Et Biophysica Acta Reviews on Cancer*.

[6] Kalluri R., LeBleu V. S. (2020). The biology, function, and biomedical applications of exosomes. *Science*.

[7] Zhang X., Sai B., Wang F. (2019). Hypoxic BMSC-derived exosomal miRNAs promote metastasis of lung cancer cells via STAT3-induced EMT. *Molecular Cancer*.

[8] Zhang J., Li S., Li L. (2015). Exosome and exosomal microRNA: trafficking, sorting, and function. *Genomics, Proteomics & Bioinformatics*.

[9] Saliminejad K., Khorram Khorshid H. R., Soleymani Fard S., Ghaffari S. H. (2019). An overview of microRNAs: biology, functions, therapeutics, and analysis methods. *Journal of Cellular Physiology*.

[10] Meng Q., Liang C., Hua J. (2020). A miR-146a-5p/TRAF6/NF-kB p65 axis regulates pancreatic cancer chemoresistance: functional validation and clinical significance. *Theranostics*.

[11] Cui Y., Zhang C., Ma S., Guan F. (2021). TFAP2A-induced SLC2A1-AS1 promotes cancer cell proliferation. *Biological Chemistry*.

[12] Jing R., Ma B., Qi T. (2020). Long noncoding RNA OIP5-AS1 promotes cell apoptosis and cataract formation by blocking POLG expression under oxidative stress. *Investigative Ophthalmology & Visual Science*.

[13] Beck A. C., Cho E., White J. R. (2021). AP-2*α* regulates S-phase and is a marker for sensitivity to PI3K inhibitor Buparlisib in colon cancer. *Molecular Cancer Research*.

[14] Liu H., Deng H., Zhao Y., Li C., Liang Y. (2018). LncRNA XIST/miR-34a axis modulates the cell proliferation and tumor growth of thyroid cancer through MET-PI3K-AKT signaling. *Journal of Experimental & Clinical Cancer Research*.

[15] Enright A. J., John B., Gaul U., Tuschl T., Sander C., Marks D. S. (2003). MicroRNA targets in drosophila. *Genome Biology*.

[16] Kern F., Aparicio-Puerta E., Li Y. (2021). miRTargetLink 2.0-interactive miRNA target gene and target pathway networks. *Nucleic Acids Research*.

[17] Chen Y., Wang X. (2020). miRDB: an online database for prediction of functional microRNA targets. *Nucleic Acids Research*.

[18] Agarwal V., Bell G. W., Nam J. W., Bartel D. P. (2015). Predicting effective microRNA target sites in mammalian mRNAs. *ELife*.

[19] Tang Z., Li C., Kang B., Gao G., Li C., Zhang Z. (2017). GEPIA: a web server for cancer and normal gene expression profiling and interactive analyses. *Nucleic Acids Research*.

[20] Liu S. H., Zhang W. G., Zhang M., Zhu Q., Tian W. (2005). Construction and identification of Fas-targeting siRNA-expressing plasmid. *Journal of Zhejiang University Science B*.

[21] Liang G., He H., Li Y., Yu D. (2012). A new strategy for construction of artificial miRNA vectors in Arabidopsis. *Planta*.

[22] Yin Z., Ma T., Huang B. (2019). Macrophage-derived exosomal microRNA-501-3p promotes progression of pancreatic ductal adenocarcinoma through the TGFBR3-mediated TGF-*β* signaling pathway. *Journal of Experimental & Clinical Cancer Research*.

[23] Kajioka H., Kagawa S., Ito A. (2021). Targeting neutrophil extracellular traps with thrombomodulin prevents pancreatic cancer metastasis. *Cancer Letters*.

[24] Xiao Y., Cong M., Li J. (2021). Cathepsin C promotes breast cancer lung metastasis by modulating neutrophil infiltration and neutrophil extracellular trap formation. *Cancer Cell*.

[25] Pegtel D. M., Gould S. J. (2019). Exosomes. *Annual Review of Biochemistry*.

[26] Mashouri L., Yousefi H., Aref A. R., Ahadi A. ., Molaei F., Alahari S. K. (2019). Exosomes: composition, biogenesis, and mechanisms in cancer metastasis and drug resistance. *Molecular Cancer*.

[27] Costa-Silva B., Aiello N. M., Ocean A. J. (2015). Pancreatic cancer exosomes initiate pre-metastatic niche formation in the liver. *Nature Cell Biology*.

[28] Mo F., Luo Y., Fan D. (2020). Integrated analysis of mRNA-seq and miRNA-seq to identify c-MYC, YAP1 and miR-3960 as major players in the anticancer effects of Caffeic acid Phenethyl Ester in human small cell lung cancer cell line. *Current Gene Therapy*.

[29] Zheng Y., Song A., Zhou Y. (2020). Identification of extracellular vesicles-transported miRNAs in Erlotinib-resistant head and neck squamous cell carcinoma. *Journal of Cell Communication and Signaling*.

[30] Xiong Y., Feng Y., Zhao J. (2021). TFAP2A potentiates lung adenocarcinoma metastasis by a novel miR-16 family/TFAP2A/PSG9/TGF-*β* signaling pathway. *Cell Death & Disease*.

[31] Zhang P., Hou Q., Yue Q. (2020). MiR-204-5p/TFAP2A feedback loop positively regulates the proliferation, migration, invasion and EMT process in cervical cancer. *Cancer Biomarkers*.

[32] Wilkins H. M., Marquardt K., Lash L. H., Linseman D. A. (2012). Bcl-2 is a novel interacting partner for the 2-oxoglutarate carrier and a key regulator of mitochondrial glutathione. *Free Radical Biology & Medicine*.

[33] Ding M., Fu Y., Guo F. (2020). Long non-coding RNA MAFG-AS1 knockdown blocks malignant progression in breast cancer cells by inactivating JAK2/STAT3 signaling pathway via MAFG-AS1/miR-3196/TFAP2A axis. *International Journal of Clinical and Experimental Pathology*.

[34] Han B., Sun Y., Yang D. (2020). USP22 promotes development of lung adenocarcinoma through ubiquitination and immunosuppression. *Aging*.

[35] Chen S., He Z., Zhu C. (2020). TRIM37 mediates Chemoresistance and maintenance of Stemness in pancreatic cancer cells via ubiquitination of PTEN and activation of the AKT-GSK-3*β*-*β*-catenin signaling pathway. *Frontiers in Oncology*.

[36] Yang B., Feng X., Liu H. (2020). High-metastatic cancer cells derived exosomal miR92a-3p promotes epithelial-mesenchymal transition and metastasis of low-metastatic cancer cells by regulating PTEN/Akt pathway in hepatocellular carcinoma. *Oncogene*.

